# Emerging ASL Distinctions in Sign-Speech Bilinguals' Signs and Co-speech Gestures in Placement Descriptions

**DOI:** 10.3389/fpsyg.2021.686485

**Published:** 2021-08-03

**Authors:** Anne Therese Frederiksen

**Affiliations:** ^1^Department of Linguistics University of California, San Diego, La Jolla, CA, United States; ^2^Department of Language Science, University of California, Irvine, Irvine, CA, United States

**Keywords:** speech-sign bilingualism, caused motion events, bidirectional language influences, sign language, co-speech gestures, iconicity, second language, fuzzy lexical representations

## Abstract

Previous work on placement expressions (e.g., “she put the cup on the table”) has demonstrated cross-linguistic differences in the specificity of placement expressions in the native language (L1), with some languages preferring more general, widely applicable expressions and others preferring more specific expressions based on more fine-grained distinctions. Research on second language (L2) acquisition of an additional spoken language has shown that learning the appropriate L2 placement distinctions poses a challenge for adult learners whose L2 semantic representations can be non-target like and have fuzzy boundaries. Unknown is whether similar effects apply to learners acquiring a L2 in a different sensory-motor modality, e.g., hearing learners of a sign language. Placement verbs in signed languages tend to be highly iconic and to exhibit transparent semantic boundaries. This may facilitate acquisition of signed placement verbs. In addition, little is known about how exposure to different semantic boundaries in placement events in a typologically different language affects lexical semantic meaning in the L1. In this study, we examined placement event descriptions (in American Sign Language (ASL) and English) in hearing L2 learners of ASL who were native speakers of English. L2 signers' ASL placement descriptions looked similar to those of two Deaf, native ASL signer controls, suggesting that the iconicity and transparency of placement distinctions in the visual modality may facilitate L2 acquisition. Nevertheless, L2 signers used a wider range of handshapes in ASL and used them less appropriately, indicating that fuzzy semantic boundaries occur in cross-modal L2 acquisition as well. In addition, while the L2 signers' English verbal expressions were not different from those of a non-signing control group, placement distinctions expressed in co-speech gesture were marginally more ASL-like for L2 signers, suggesting that exposure to different semantic boundaries can cause changes to how placement is conceptualized in the L1 as well.

## Introduction

In learning how to say the equivalent of “the woman put the cup on the table” in a second language, many learners face the challenge of semantic reconstruction. A speaker whose first language (L1) is English and whose second language (L2) is Dutch must learn that Dutch does not have one verb that corresponds to “put” in English. Instead, when describing an event of putting in Dutch, the speaker must choose between different verbs. This choice requires attention to the shape and orientation of the object being placed. Thus, the learner must not only learn the appropriate vocabulary in the target language but may also need to reorganize their conceptualization of placement events. This is a challenge for many learners whose tendency to transfer semantic boundaries from the L1 onto the L2 can result in non-target like use of verbs of placement, indicating fuzzy placement semantics. Unknown is whether differences in semantic transparency in the target-language may help acquisition. Like spoken languages, sign languages use different verbal distinctions in descriptions of placement of different objects. Unlike spoken language verbs in many languages, however, placement verbs are often highly iconic in sign languages. They involve handshapes reflecting visual properties of their referents, and/or kinesthetic properties of how an entity is handled. Placement descriptions in sign languages therefore offer a transparent link between elements of the world and their linguistic encoding. It is unknown whether such transparency facilitates acquiring placement expressions for hearing second language learners of a sign language, or whether they experience the same difficulties in acquiring novel, semantic distinctions as do hearing second language learners of a spoken language.

Also poorly understood are the consequences for L1 placement semantics of learning a typologically different L2. The process of acquiring target-like semantic boundaries may require the learner to engage in semantic reconstruction. As the L2 is fully or partially acquired, this process may come to influence the L1, creating a system where the semantic boundaries of the L1 are (temporarily) fuzzy and unstable and consequently may differ from that of monolinguals and bilinguals with a different L2.

The present study aims to address these gaps in our knowledge by investigating placement descriptions in native English speakers learning American Sign Language (ASL) as an L2. Placement expressions are highly transparent in American Sign Language and at the same time, they exhibit some form overlap with co-speech gestures used in placement descriptions. We take advantage of these facts to ask (1) whether acquiring target-like placement verbs is challenging for different-modality L2 learners as has been shown for same-modality learners, or whether the transparency of ASL placement verbs decreases the difficulty of this task, and (2) whether the learners' English placement descriptions (speech and gesture) show evidence of influence from ASL.

### Placement Events

Languages show considerable differences in the expression of placement events (Kopecka and Narasimhan, 2012). A placement event is a type of caused motion event, in which an agent moves something somewhere, e.g., putting a book on a bookshelf. Studies have shown that the descriptions of placement is a typologically quite diverse domain cross-linguistically, not least in terms of verb semantics (see Bohnemeyer and Pederson, 2011; Gullberg, 2011a; Slobin et al., 2011; Kopecka and Narasimhan, 2012). This is perhaps surprising. Given that speakers from different cultures share similar visual and motor experiences with respect to placing objects, we might expect them to describe those experiences in similar ways. However, studies from the last decades have shown that this is far from the case (Ameka and Levinson, 2007; Bohnemeyer and Pederson, 2011; Kopecka and Narasimhan, 2012). Narasimhan et al. (2012) note that languages such as Hungarian, Kalasha, Hindi, and Tamil use a semantically general verb for “put” (as do languages like English and French). This type of single-term or general placement verb language is in opposition to multi-term or specific placement verb languages, such as Tzeltal, which requires selection of one of numerous verb roots to describe a placement event. Languages such as Dutch, Swedish, Polish, and Yeli Dnye are a slightly different kind of multi-term languages. They select a verb from small set of so-called posture verbs, depending on several factors, including the orientation of the object being placed. For example, German, also a posture verb language, distinguishes between the verbs “*stellen”* “to put upright,” “*setzen”* “to set,” “*legen”* “to lay,” “*stecken”* “to stick,” and “*hängen”* “to hang” (De Knop, 2016).

**Figure 1:**
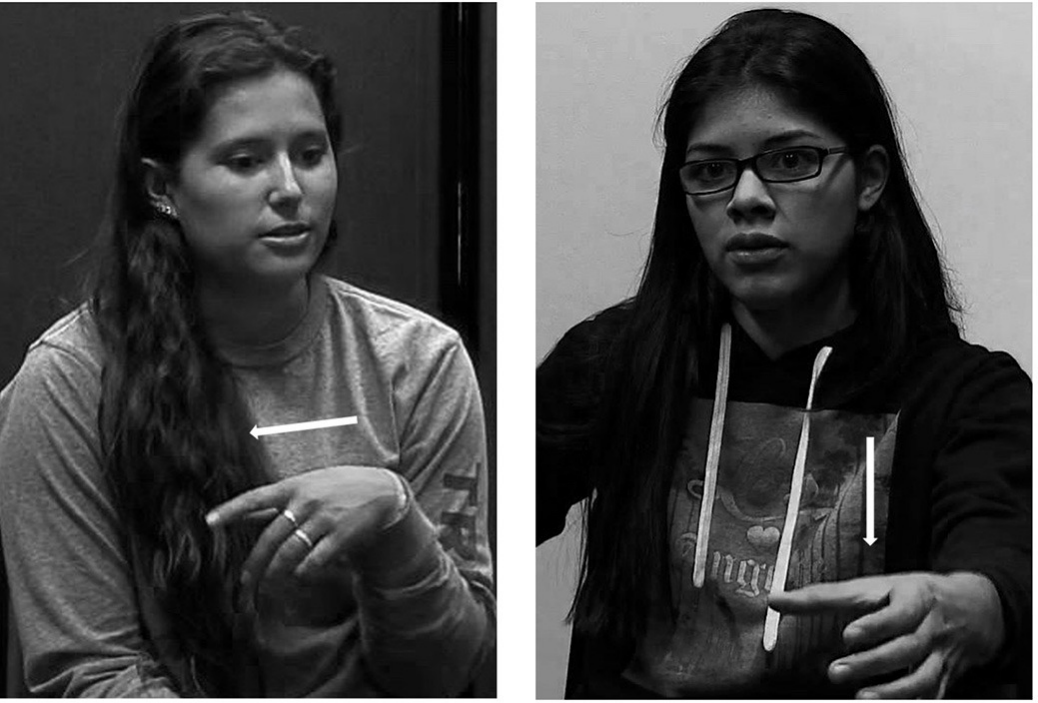
Path gesture in description of placing a clothes hanger (left) and figure + path gesture in description of placing a speaker (right).

Relevant semantic distinctions show up in the co-speech gestures of a language, as well as in speech (Hoetjes, 2008; Gullberg, 2009a, 2011a). Speakers frequently accompany their words with co-speech gesture (McNeill, 1992; Kendon, 2004). Many co-speech gestures are iconic representations of some part of the speech content, that is, they are handshapes that share form properties with the represented entity or action (Kendon, 1980; McNeill and Levy, 1982; McNeill, 1992). Because placement descriptions denote placement actions, which are similar across languages, we might expect speakers to accompany their descriptions with similar co-speech gestures irrespective of the variation in semantic distinctions in different languages. For example, gesturers across languages might use handshapes similar to the motor actions used to perform placement of different items, e.g., a “cup” handshape when talking about the placement of a cup or a glass, but a pincer handshape for small objects, such as beads or coins. However, research has shown that cross-linguistic differences in placement events extend beyond the verbal component of language, and that co-speech gestures instead exhibit patterns specific to language or language type (Hoetjes, 2008; Gullberg, 2009a, 2011a). In particular, speakers of general placement verb languages like French tend to use gestures that reflect the focus in their verbal expression on the act of moving something. This means that they gesture mainly about the direction or path of an object being moved. Conversely, speakers of multi-term, specific placement verb languages like Dutch who have to select verbs in part based on properties of the object being placed typically use gestures that represent form properties of the figure object (Gullberg, 2011a). Figure 1 shows an example of a general placement verb system type path gesture compared to a specific placement verb system type gesture including both information about the figure object and the path of the placement event.

### Expression of Placement Events in English and American Sign Language

English tends to encode placement with the verb “put.” Although English has specific placement verbs based on posture (“set” and “lay”), they are infrequent in placement descriptions (Pauwels, 2000) and English is categorized as a general placement verb language. Hoetjes (2008) examined English speakers' placement verbs and placement gestures. She found that English speakers as a group used “put” in 59% of their placement descriptions, and “place,” another general placement verb, in 10% of their descriptions. In another experiment using the same stimuli, Gullberg (2009a) similarly found the mean proportion of “put” to be 61% for native English speakers. In both studies, the mean percentage of gestures incorporating information about the figure object was below 40% for native English speakers, and correspondingly, the proportion of path-only gestures was over 60%.

**Figure 2:**
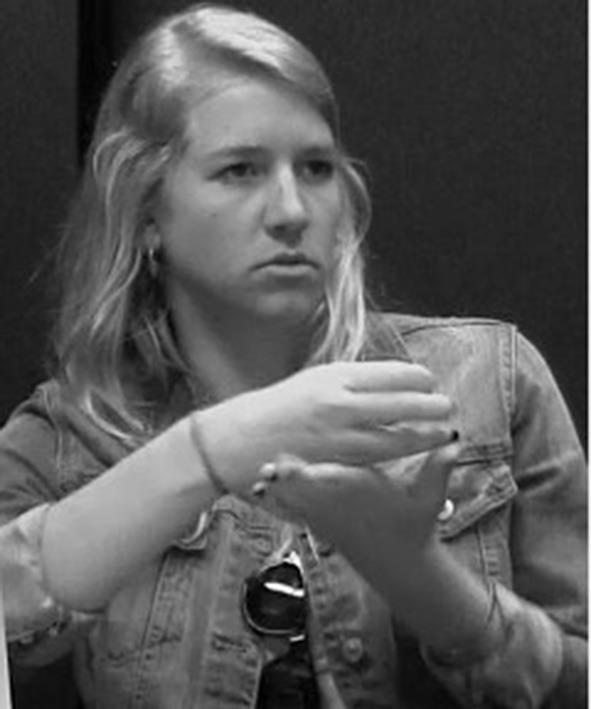
ASL Sign CUP.

To date, no studies have specifically examined placement verbs in a sign language. American Sign Language (ASL) is the primary language of most Deaf individuals in the U.S. and parts of Canada. ASL is produced with the hands and body and perceived with the eyes. Expressing language in the visual-manual modality appears to afford a high degree of iconicity (Perniss et al., 2010). For example, the ASL sign “CUP” involves a sideways C-handshape, similar to the handshape one would use to hold a cup (Figure 2). In this paper, we use capitalized letters to indicate sign glosses and letters/numbers to indicate handshapes (the relevant handshapes are pictured in the Supplementary Images). Following convention, ASL signs are glossed with English words, but note that sign and gloss are not always translation equivalents.

**Figure 3:**
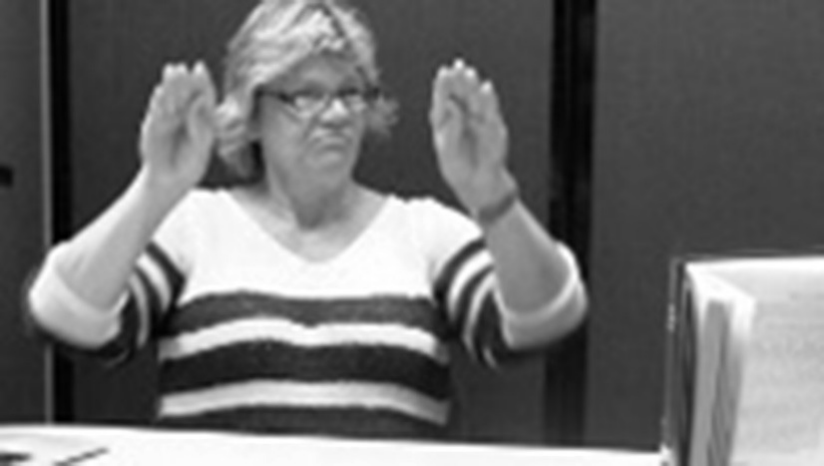
Handling classifier representing an agent holding a tablecloth.

**Figure 4:**
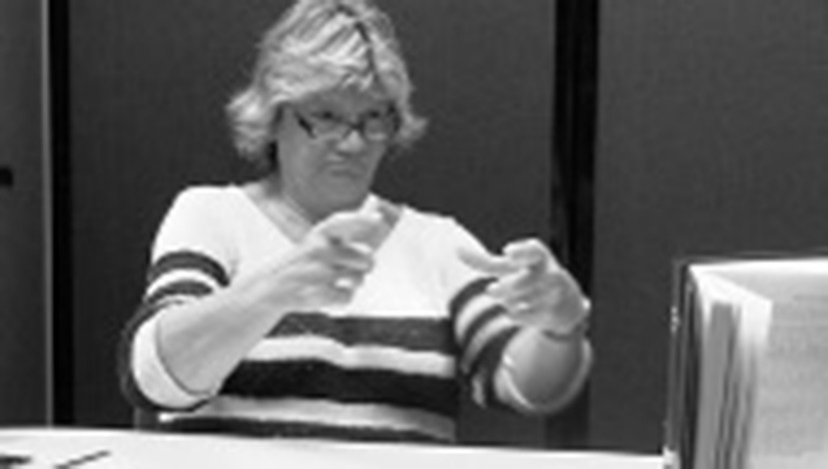
Entity classifier representing plates.

While no studies have looked specifically at how placement is expressed in ASL, some aspects of ASL and other sign languages that are relevant for understanding signed placement verbs have been investigated in previous work. Specifically, most sign languages have a system of *classifiers* (Aronoff et al., 2005; Zwitserlood, 2012) that play an important role in this domain. Classifiers are handshapes that represent something about the object being described, e.g., shape and size, semantic class, or how an agent would handle the object. There are two broad categories of classifiers (Zwitserlood, 2012): handling (or *handle*) classifiers, where the hand(s) represent(s) how the entity is held by an agent (Figure 3), and entity classifiers, where the hand(s) represent(s) the entity (Figure 4).

Although classifiers represent information about the figure object, it is not the case that there are unlimited gradient distinctions in classifiers; rather, there exists a set of conventionalized handshapes, where each is conventionally used for specific types of objects. For example, the C handshape handling classifier is used for tall cylinder-like objects like vases, cups, bottles, etc., and the flattened O handshape handling classifier is used for thin flat objects like books, papers, blankets, etc. (Zwitserlood, 2012). Both handling and entity classifiers can be incorporated into verbs of motion and location, sometimes called classifier verbs or classifier predicates (Supalla, 1982; Aronoff et al., 2003, 2005). Verbs like “PUT” and “MOVE” are examples of verbs that can be classifier verbs (e.g., Slobin et al., 2003; Slobin, 2013) and can be used in placement descriptions^1^. When describing how objects are used or manipulated, ASL signers tend to incorporate handling classifiers, rather than entity classifiers into verbs (Padden et al., 2015). Thus, ASL appears to prototypically express placement events with verbs like “MOVE” and “PUT” with incorporated handling classifiers. Despite being similar to languages like English and French in using general placement verbs such as “MOVE” and “PUT” as the basic verb, ASL is differentiated from these languages by the frequent incorporation into the verb of an additional morpheme (the classifier) which specifies shape and orientation of the figure object. Because of this, ASL can be considered a specific placement verb language^2^. We confirmed this with data from two Deaf, native ASL signers, which will be described in more detail below.

### Placement Events in the Context of L2 Acquisition

Expressing placement in a second language (L2) not only requires learning placement verbs, locative expressions and appropriate syntax. It also requires learning the semantic boundaries of placement words, which can differ, even between words that are cognates across languages. Learning new semantic boundaries requires first detecting the relevant difference and then mentally rearranging concepts and shifting boundaries accordingly. Rearranging concepts resulting from the semantics of the first language (L1) poses a challenge for the learner (Ijaz, 1986; Kellerman, 1995). This is because native language learning habituates the individual to thinking in ways that are compatible with available means of expression, i.e., to what Slobin (1996) calls “thinking for speaking.” To become target-like in placement descriptions, many L2 learners must therefore learn a new way of categorizing semantically. This can cause a variety of issues in the L2, including L1 transfer, that is, mapping semantic boundaries from the native language onto the L2, and fuzzy semantic boundaries. Non-native patterns can arise both when going from a more general to a more complex placement verb system and vice versa (Cadierno et al., 2016). When the L1 uses a general placement verb (e.g., “put” in English) and the L2 distinguishes between several specific placement verbs (e.g., “zetten” vs. “leggen” in Dutch), learners overuse one verb and do not maintain obligatory semantic distinctions (Viberg, 1998; Gullberg, 2009a). In cases where the L1 has several, specific placement verbs, and the L2 has one or more general placement verbs, learners' use of placement verbs in speech may show native-like overall distinctions relatively quickly (Gullberg, 2009a, 2011b; Lewandowski and Özçalişkan, 2021) but can nevertheless include non-native verb forms (Cadierno et al., 2016) and overuse of more peripheral verbs in an attempt to re-create placement distinctions from the L1 (Gullberg, 2009b).

Co-speech gestures have been used as a means to probe L2 speakers' underlying representations in the placement domain (Gullberg, 2009a,b, 2011a,b). While L2 learners of a more general placement verb system (e.g., Dutch L1-French L2) may be able to acquire the verbal system with relatively little difficulty, studying their gestures reveals a somewhat different picture. Using a French native-like pattern in speech does not necessarily mean that the learners have abandoned the conceptualization of placement events from their L1. Specifically, native French speakers primarily accompany their placement verbs with path-only gestures. In contrast, many L1 Dutch learners of L2 French use significantly more figure gestures, maintaining the distinctions from their native Dutch (Gullberg, 2009b), even as they are using a target-like system in their spoken French (see also the study by Özçalişkan, 2016 showing persistent L1 co-speech gesture in L2 expression of voluntary motion). Conversely, L1 speakers of a general placement verb language (e.g., English) learning a specific placement verb L2 (e.g., Dutch) produce mainly English-like path-only gestures in their L2, even when they begin to use the appropriate verb distinctions in speech (Gullberg, 2009a). Importantly, learners of a specific system do not gesture about figure-objects unless they apply the relevant distinctions in speech (Gullberg, 2009b). A general observation is that although it is difficult, and the progress is gradual, semantic reconstruction away from fuzziness and into alignment with the L2 seems possible (Gullberg, 2009b).

While evidence suggests that acquiring semantic distinctions in an L2 is challenging, it is important to note that this evidence is based exclusively on research with same-modality L2 learners, that is, individuals with a spoken L1 learning a spoken L2. However, not all second language learning happens within the same modality. Many hearing individuals acquire a sign language as an L2. Researchers are increasingly asking to what extent the principles of L2 acquisition apply when the source language is spoken and the target language is signed (Chen Pichler and Koulidobrova, 2016). While many challenges are similar for hearing learners of signed and spoken second languages, additional issues arise when acquiring a new language in a new modality (McKee and McKee, 1992; Wilcox and Wilcox, 1997), including learning to manage visual-manual phonology (Bochner et al., 2011; Chen Pichler, 2011; Ortega, 2013; Ortega and Ozyurek, 2013; Ortega and Morgan, 2015), multiple articulators (Gulamani et al., 2020), spatial grammar and depicting referents with the body (Bel et al., 2014; Ferrara and Nilsson, 2017; Frederiksen and Mayberry, 2019; Kurz et al., 2019; Gulamani et al., 2020), using the face to display grammatical information (McIntire and Reilly, 1988), and the high degree of iconicity in sign languages (Lieberth and Gamble, 1991; Campbell et al., 1992; Baus et al., 2013; Ortega and Morgan, 2015). At the same time, it is possible that hearing learners' experience with co-speech gesture in their L1 affects their acquisition of a sign language (McIntire and Reilly, 1988; Taub et al., 2008; Chen Pichler and Koulidobrova, 2016). Hearing individuals produce spontaneous co-speech gestures when they speak. However, it is as of yet unclear whether co-speech gesture in fact helps or hinders sign acquisition, and previous work suggests that the answer to this question may vary by linguistic domain (Schembri et al., 2005; Ortega and Morgan, 2010; Chen Pichler, 2011; Ortega, 2013; Marshall and Morgan, 2015; Janke and Marshall, 2017; Kurz et al., 2019).

To date, it is unknown whether the acquisition of placement expressions is similarly difficult when acquiring a signed compared to a spoken L2. Many researchers have noted similarities between the classifier handshapes used by Deaf signers and the handshapes used in co-speech gesture and pantomime by hearing individuals in (Singleton et al., 1993; Schembri et al., 2005; Sevcikova, 2014; Marshall and Morgan, 2015; Quinto-Pozos and Parrill, 2015), despite obvious differences such as signers tapping into a much more conventionalized system and gesturers employing these handshapes on the fly. Other studies show similarities in how the signers and gesturers alternate between different (classifier) handshapes in their descriptions of objects and humans handling them (Brentari et al., 2012; Padden et al., 2015; Masson-Carro et al., 2016; Hwang et al., 2017; van Nispen, 2017; Ortega, 2020). Thus, it is possible that English speakers can build on their use of gestural distinctions to acquire ASL semantic boundaries in placement verbs relatively quickly. Moreover, the high degree of transparency in ASL placement distinctions may also be an advantage for L2 learners, decreasing the proficiency level required to use target-like placement distinctions in ASL compared to learners acquiring a less transparent system.

### Bidirectional Language Influences

Research has shown that language influence can happen in both directions, from L1 to L2 but also from L2 to L1. In bilinguals, the two languages are activated at the same time and compete for selection (e.g., Jared and Kroll, 2001; Marian and Spivey, 2003; Dijkstra, 2005; Kroll et al., 2008). This not only results in influence from the first on the second language; even at the very beginning stages, learning a second language affects the first language (see Kroll et al., 2015). Further, this effect is not only observed with respect to language processing, but also in how events are conceptualized, e.g., “conceptual transfer” (Bylund and Jarvis, 2011; Daller et al., 2011). Bi-directional effects have been observed in the context of cross-modality language learning as well. Work by Morford et al. has shown that ASL signs are activated during English print word recognition in highly proficient ASL-English bilinguals, irrespective of language dominance (Morford et al., 2011, 2014). Similar effects have been reported for DGS (Deutsche Gebärdensprache, *German Sign Language*)-German bimodal bilinguals (Kubus et al., 2015; Hosemann et al., 2020).

L2 influence on placement verb semantic boundaries in L1 has not been researched specifically for the case when the L1 is clearly dominant and the L2 is weaker. However, Alferink and Gullberg (2014) investigated placement verbs in individuals who grew up with and continued to use both French and Dutch in their daily lives. These early bilinguals showed evidence of having blurred obligatory placement verb distinctions in Dutch, effectively using the same distinctions for both Dutch and French despite the former being a specific, multi-term language and the latter being a general, single-term language.

Thus, there is reason to expect L2 learners' placement expressions in English to be influenced by ASL. Such an influence could be evident in either speech or in gesture. Co-speech gesture research has found evidence of gestural transfer from the L2 to the L1 (Brown and Gullberg, 2008, 2011; see also overview in Gullberg, 2009c). Specifically, in L1 descriptions of voluntary motion, some L2 learners show evidence of aligning with the L2 in gesture while maintaining L1 patterns in speech. Pertinent to the present study, there appears to be an additional effect on L1 co-speech gesture from learning a signed as opposed to a spoken L2. Iconic gesture rates increase with sign language proficiency, something that does not happen when learning spoken second languages, even in languages known for frequent gestures, such as French or Italian (Casey et al., 2012; Weisberg et al., 2020). For hearing English-ASL bilinguals, there is the additional factor that classifiers, and particularly classifiers of the *handling* type that are predominant in placement descriptions, have iconic properties reflecting visuo-spatial properties of their referents (Zwitserlood, 2012), which offers a visual correspondence between elements of the world and their linguistic encoding. It has been shown that, in some domains, co-speech gesturers and signers tend to use their hands in similar ways (Sevcikova, 2014; Quinto-Pozos and Parrill, 2015). As such, placement descriptions in sign languages offer a visual correspondence between elements of the world and their linguistic encoding which may be easy to adopt either because it already overlaps with the distinctions used by the learners in co-speech gesture, or because of the high degree of transparency in the distinctions that are being employed in ASL.

## The Present Study

The present study investigates what semantic reorganization in the domain of placement events looks like when the source and target languages do not share a sensory-motor modality. We ask two major questions: First, whether second language learners of ASL face similar challenges with placement verbs as do same-modality L2 learners, especially in the light of the high degree of transparency in ASL placement verb distinctions. Second, we ask whether there is evidence that learning new semantic boundaries for placement events affects L1 semantics. If modality and transparency do not matter, then we would expect English L1-ASL L2 language users to use general placement verbs such as “PUT” and “MOVE,” and specifically, L2 signers should use classifiers at a lower rate than native signers and exhibit fuzzy semantic boundaries for the classifiers they do use. Further, if L1 semantics is affected by learning a signed L2, then we would expect L2 signers' placement descriptions in English speech and/or co-speech gesture to include ASL-like distinctions that do not occur in non-signing English speakers. Specifically, L2 signers would be expected to use comparatively more verbs with a specific rather than general placement meaning, and/or to use more co-speech gestures reflecting properties of the figure object.

### Methods

#### Participants

We recruited eight hearing L2 signers (five female) to take part in the task. These individuals were native speakers of English who learned ASL as young adults. All were intermediate learners who had completed at least 1 year of ASL instruction (six weekly contact hours). At the time of participation, seven of the learners used ASL daily, and one learner used ASL once a month. Seven of the L2 learners had exposure to either Spanish or French starting between the ages of nine and fourteen. Table 1 summarizes their demographic information. We additionally tested eight non-signing English speakers (five female, mean age: 19; SD: 1), and two Deaf, native ASL signers (two female, ages 21 and 62), on the same task. Seven of the non-signers had exposure to a language other than English (Spanish, French, Farsi, German); three were exposed to the non-English language after the age of 11, two at the age of eight, and two were exposed to Spanish from birth ^3^. All non-signing participants reported that they were English dominant.

**Table 1:** The L2 signers' background information.

**Participant information**	**Mean**	**Range**	**SD**
Age (years)	21	19–22	1
Age began ASL learning (years)	18	13–20	2
Time learning ASL (years)	2.88	1–8	2.30
Self-reported expressive ASL proficiency (of 10)	6.75	4–9	1.49

#### Stimuli and Procedure

**Figure 5:**
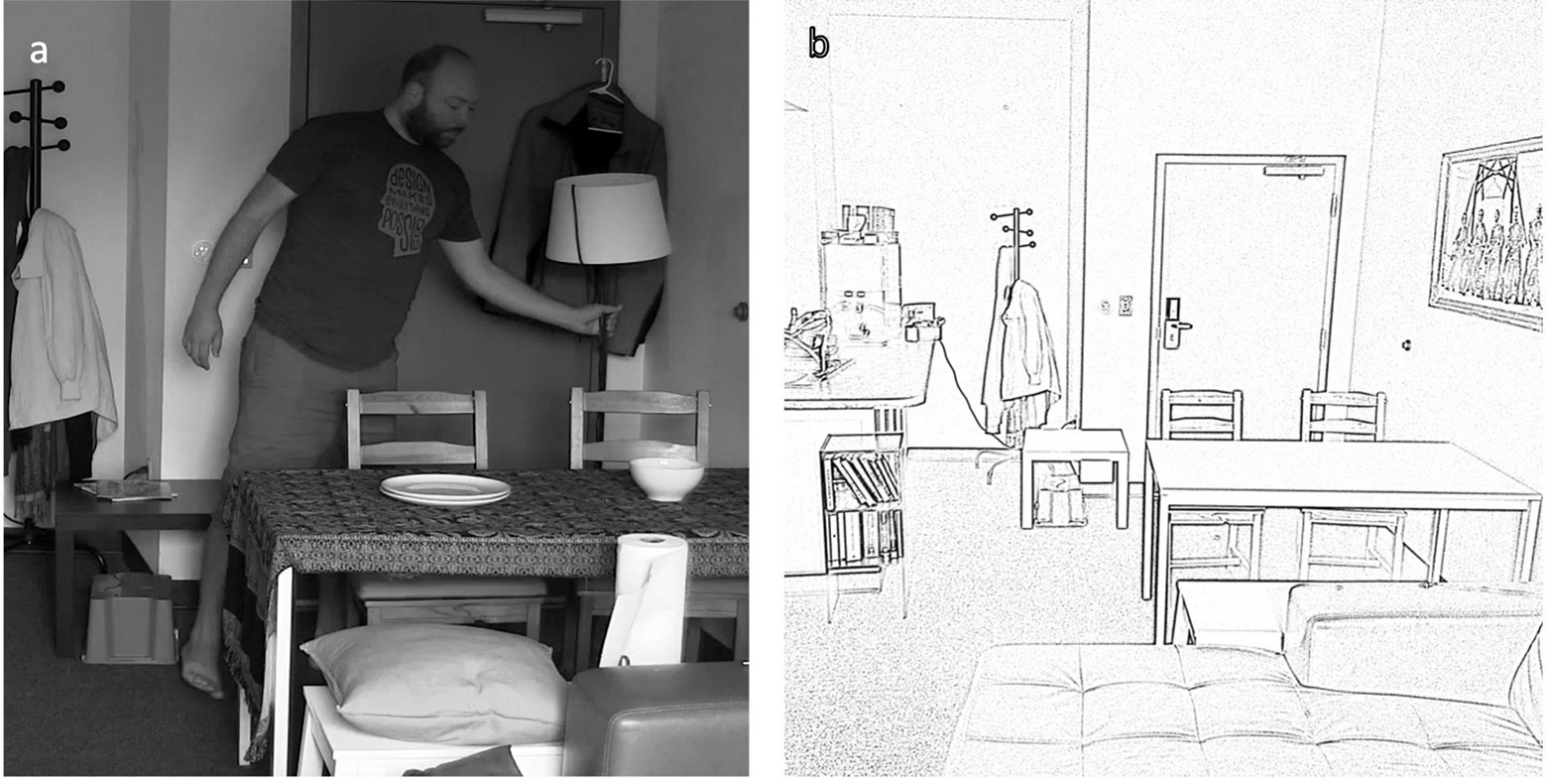
Still picture from a stimulus clip **(a)** and picture for the matcher's drawing **(b)**.

We used a director-matcher task (e.g., Clark and Wilkes-Gibbs, 1986) to elicit placement descriptions of our stimuli. The stimuli consisted of a video segment showing a man repositioning objects in a room. The video was split into six parts, each containing the placement of four or five stimulus objects (e.g., a cup, a lamp, plates, a scarf), for a total of 25 events (see Appendix in Supplementary Material for a full list of stimulus items). In our version of the director-matcher task, the participant was the director, and their task was to watch the video clips (Figure 5a) one at a time and explain to the matcher (a native language user confederate) what happened after each clip. The matcher in turn drew this information on a picture of the empty room Figure 5b), specifically where the objects being described were placed. The video clips were not visible to the matcher and the drawing was not visible to the director.

After providing written, informed consent, the director and matcher were seated across from each other, and the experimenter explained their tasks in the language of the experiment. As a memory aid, the director was given list of pictures corresponding to the objects-to-be-described^4^. The matcher was instructed to ask questions whenever clarification was needed. Two cameras captured the communication between the director and the matcher during the task. Only data from the director are analyzed in this study. The L2 signers completed the task twice, once in English and once in ASL with different interlocutors. We counterbalanced the order of languages, and participants performed another task before completing the task the second time. Participants were either paid a small amount or given credit in a college course for their participation.

#### Transcription and Coding

Speech and sign transcriptions were done in the ELAN software (Wittenburg et al., 2006) by transcribers who were native language users. For each of the stimulus objects, they identified the first complete, spontaneous, and minimal placement description. Such descriptions included mention of the figure object, generally in the form of a lexical noun phrase, the verb (or intransitive construction, e.g., “the cup is on the table”), and often the final location of the object as well. Repetitions, elaborations and answers to questions by the interlocutor were not included. From each of the included descriptions, the transcriber provided a verbatim transcription of the placement event specifically, shown in italics in 1) for English and 2) for ASL.

1. *[then he grabbed the paper towels] and* placed *them on the kitchen counter*
2. [BOOK TAKE-handshape: C] *CABINET* PUT-handshape: C *[(he) took the book] (and)* put *it in the cabinet* 
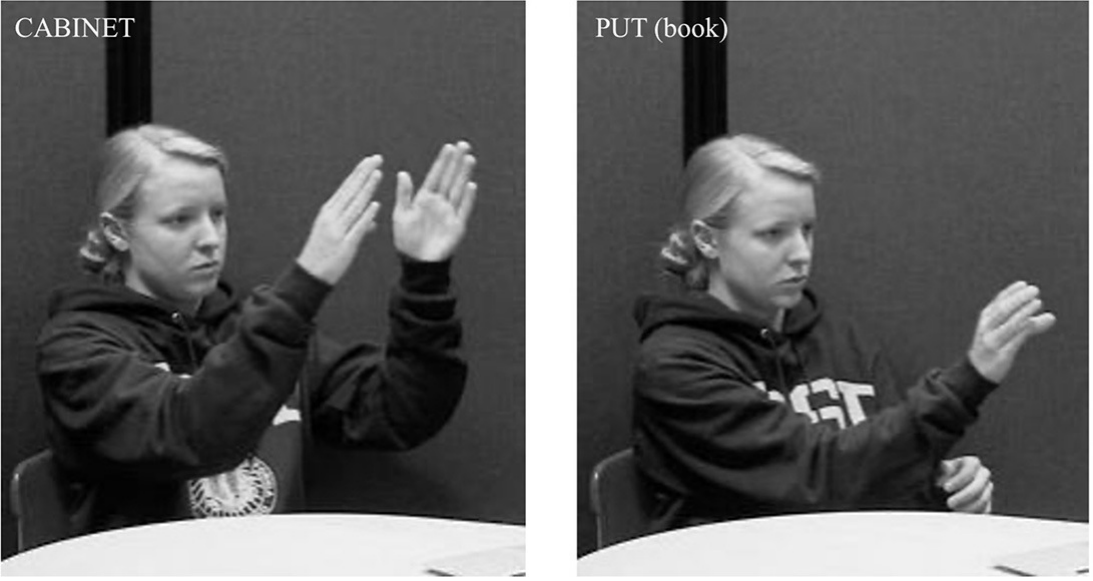

Finally, it was noted which placement verb was used in each placement description [underlined in (1) and (2)]. For ASL trials, two trained coders, a Deaf native signer and a hearing proficient signer, noted whether the placement verb was used in the citation form or with an incorporated classifier. The coders were instructed to be conservative when encountering handshapes that were ambiguous between citation form and incorporated classifier. For example, holding a thin flat object in a horizontal position would use the same flat O handshape as the citation form of “PUT.” In such cases, the verb was coded as occurring in citation form unless the hand orientation or movement was different from the citation form. Flat and open/round versions of the same handshape were grouped together. Where applicable, the coders also noted the type of classifier (handling vs. entity) used in the placement verb. This resulted in three categorizations of verbs and intransitive constructions with possible classifier incorporation: (1) Handling classifier, (2) Entity classifier, and (3) Citation form. Across the entire data set, the two coders agreed on 92% of categorizations.

For gesture transcriptions, we focused on tokens occurring during the minimal placement descriptions identified for speech. We marked gesture strokes, that is, the most effortful and expressive part of the gesture, and post-stroke holds, that is, periods of maintaining the stroke handshape after the stroke itself (Kita et al., 1998; Kendon, 2004). For each gesture identified, two coders separately noted whether the handshape and hand orientation (a) expressed figure information by reflecting properties of the stimulus object in question, (b) did not reflect figure object properties but only indicated direction or path of movement, (c) reflected only properties of the ground object on which the figure object was placed, or (d) did not have any relationship to the placement event proper, that is, the gesture was a beat gesture serving to emphasize speech rhythm, a thinking gesture indicating word finding difficulty, or it was unclear what the gesture represented. This resulted in the following gesture categories: (1) Figure inclusion (a), (2) Path only (b), (3) Everything else (c and d). To minimize influence on gesture coding from the speech content, this coding was undertaking without access to the video's sound. Across the entire data set, the two coders agreed on 90% of form categorizations. In cases of disagreement, the judgement of the first coder was retained.

## Results

The analysis focuses on the target objects for which the placement description was complete. Some trials were skipped, most likely because participants simply forgot to mention a target object. Non-signing participants skipped nine target objects (5%), native ASL signers skipped one target object (2%), and the L2 ASL learners skipped ten (5%) target objects in English and 15 (7.5%) in ASL.

### ASL

We first confirmed that the Deaf, native signer control data matched our expectations of ASL as using primarily general verb types like “PUT,” and “MOVE” modified with classifiers to reflect properties of the figure object (see verb illustrations in the Supplementary Images). As shown in Table 2, the verb types “MOVE” and “PUT” together accounted for 88% of verb tokens used. “HANG” was the only additional verb used with any regularity. The native signers' mean proportion of verbs incorporating a classifier handshape was 66%. The vast majority of classifiers were handling classifiers (84%); only a small proportion were entity classifiers (16%). Table 3 shows how often different verbs were produced with classifiers. On average, “MOVE” was used with a classifier more than half the time. 92% of those classifiers were handling classifiers. The verb “PUT” never occurred in citation form in these data; it was used with a handling classifier 94% of the time. Overall, seven different classifiers occurred: three handshapes occurred as handling classifiers (O, C, S) and four occurred as entity classifiers (Y, B, C, baby-C; see illustrations of classifier handshapes in the Supplementary Images). Thus, while a few non-specific placement verbs account for the vast majority of tokens in native ASL signers' placement descriptions, they are modified with classifiers more often than not, which creates a complex system involving multiple distinctions based on properties of the figure object.

**Table 2:** Verb types by group.

	**Native signers**		**L2 signers**	
	**Mean% (*N*)**	**SD**	**Mean% (*N*)**	**SD**
MOVE	0.67 (33)	0.24	0.35 (64)	0.29
PUT	0.21 (10)	0.24	0.32 (59)	0.13
HANG	0.08 (4)	0.06	0.08 (15)	0.05
DRAPE	0.02 (1)	0.03	0.02 (3)	0.03
Intrans.	0.02 (1)	0.03	0.18 (33)	0.21
SET	0.00 (0)	-	0.06 (10)	0.09

**Table 3:** Classifier incorporation by verb and group^5^.

	**Native signers**		**L2 signers**	
	**Mean% (*N*)**	**SD**	**Mean% (*N*)**	**SD**
MOVE	0.62 (20)	0.07	0.65 (28)	0.45
PUT	1.00 (10)	0.00	0.87 (54)	0.23
HANG	0.17 (1)	0.24	0.92 (14)	0.20
DRAPE	1.00 (1)	-	1.00 (3)	0.00
Intrans.	NA	NA	0.09 (5)	0.18

We next asked whether the L2 signers similarly used general placement verbs modified with classifiers in their placement descriptions, which would suggest they that have reconstructed their conceptualization of placement events and are using semantic distinctions that are relevant in the target language.

An examination of how the L2 learners' use of broad ASL verb types compares with that of the two Deaf, native signers, irrespective of classifier use, shows that the L2 signers use “MOVE” and “PUT” most frequently, similarly to the native signers (Table 2). However, where the native signers use a greater proportion of “MOVE,” L2 signers on average use the two verbs at a similar rate. The main difference between the groups' overall verb use is the L2 learners' use of a large proportion of intransitive descriptions, and their use of “SET,” which are low or absent from the native data^6^. Both groups use comparable proportions of “HANG” and “DRAPE.” Despite differences in proportion, an analysis of variance on the proportion of tokens (arcsine transformed) as a function of verb type and group showed no difference in the use of broad ASL verb types by the L2 learners as compared to the native signers [*F*_(6, 48)_ = 1.656, *p* = 0.153].

^5^

^6^

We next asked whether L2 and native signers incorporated classifiers into their verbs at similar rates. Aggregating across verbs, the mean rate of classifier incorporation was 56% (SD = 27) for L2 signers, compared with 66% (SD = 13) for native signers. Nevertheless, a mixed-effect logistic regression (Jaeger, 2008) showed no significant effect of group (ß = −0.329, *p* = 0.77)^7^. Table 3 shows how often classifiers were incorporated into the different verbs by native and L2 signers. Overall, the groups incorporated classifiers similarly. On average, L2 signers used a high rate of classifiers for both “MOVE” and “PUT,” which is similar to the native signers. The main difference was in the verb “HANG,” where L2 signers incorporated classifiers at a much higher rate than native signers. As for classifier type, the L2 signers were similar to the native signers in using mostly handling classifiers (79%), with fewer entity classifiers (21%). The learners used both “MOVE” and “PUT” primarily with handling classifiers. Overall, nine classifiers occurred in the L2 data: four handshapes were used as entity classifiers (C, B, baby-C, F) and five were used as handing classifiers (A, 5, C, O, S). The native signers used the handshapes C, B, and baby-C as well as Y as entity classifiers. The two groups overlapped in their use of the handling classifier handshapes C, O and S, and the L2 signers additionally used A and 5 handshapes.

We finally asked whether the classifiers used by the L2 signers were appropriate for the described objects, as compared with the native signers. We grouped the described objects into five categories based on shared characteristics that were expected to influence how placement of the object would be expressed: (1) Tall cylindrical objects with a thin handle or neck (wine bottle, water bottle, paper towel holder, lamp, and candle), (2) Objects with a functional base (glass, potted plant, plates, speaker, basket, and bowl), (3) Thin rectangular objects without clear functional base (picture frame, magazine, computer, and book), (4) Object made from fabric (jacket, table cloth, pillow, scarf, throw, bag, and hat), and (5) Other (cables, silverware, clothes hanger)^8^. We then examined which handshapes were preferred by native and L2 signers for objects in each of the five categories (Table 4). Visual inspection of Table 4 shows that, excepting the Fabric category, the L2 signers used more verbs without classifiers than the native signers for all categories^9^. The most frequently occurring classifier was the same in both groups for the categories Tall Cylinder, Functional Base and Thin Rectangle. At the same time, the L2 signers used the preferred classifier in each group at a numerically lower rate than the native signers. Moreover, the L2 signers' use of classifiers was more variable than the native signers' across all categories.

**Table 4:** Classifier types by group and object type.

		**Native signers % (N)**	**L2 signers % (N)**
Tall cylinder	Handling		
	S	1.00 (10)	0.256 (10)
	C	0.00 (0)	0.103 (4)
	A	0.00 (0)	0.077 (3)
	O	0.00 (0)	0.026 (1)
	Entity		
	B	0.00 (0)	0.026 (1)
	F	0.00 (0)	0.026 (1)
	SET	0.00 (0)	0.179 (7)
	No classifier	0.00 (0)	0.308 (12)
Functional base	Handling		
	C	0.583 (7)	0.261 (12)
	S	0.00 (0)	0.043 (2)
	5	0.00 (0)	0.022 (1)
	O	0.00 (0)	0.022 (1)
	A	0.00 (0)	0.022 (1)
	Entity		
	Baby-C	0.083 (1)	0.13 (6)
	No classifier	0.333 (4)	0.50 (23)
Thin rectangle	Handling		
	C	0.25 (2)	0.167 (5)
	O	0.25 (2)	0.033 (1)
	A	0.00 (0)	0.067 (2)
	Entity		
	B	0.125 (1)	0.133 (4)
	Baby-C	0.00 (0)	0.033 (1)
	SET	0.00 (0)	0.100 (3)
	No classifier	0.375 (3)	0.467 (14)
Fabric	Handling		
	O	0.154 (2)	0.08 (4)
	S	0.077 (1)	0.08 (4)
	A	0.00 (0)	0.40 (20)
	C	0.00 (0)	0.08 (4)
	5	0.00 (0)	0.02 (1)
	Entity		
	Y	0.077 (1)	0.00 (0)
	C	0.077 (1)	0.06 (3)
	B	0.00 (0)	0.02 (1)
	No classifier	0.615 (8)	0.26 (13)
Other	Handling		
	O	0.167 (1)	0.00 (0)
	S	0.333 (2)	0.10 (2)
	A	0.00 (0)	0.35 (7)
	5	0.00 (0)	0.15 (3)
	Entity		
	Y	0.167 (1)	0.00 (0)
	No classifier	0.333 (2)	0.40 (8)

We asked whether the L2 signers' classifier handshapes were appropriate for the figure objects in question. A Deaf, native ASL signer rated the appropriateness of classifier handshapes produced by each signer for each target object on a scale from 1 to 5. A rating of 1 corresponded to “very bad ASL,” and a rating of 5 corresponded to “very good ASL.” Submitting these ratings to an ordinal mixed effects model^10^ revealed a significant difference between groups (L.R. χ^2^ = 4.4711, *df* = 1, *p* < 0.05), with native signers receiving higher ratings (M = 4.78, SD = 0.04) than the L2 signers (M = 3.19, SD = 1.05).

### English

#### English Verbs

To assess whether the L2 signers experience influence from ASL on their L1, English, we first asked whether they use English placement verbs similarly to non-signers. As shown in Table 5, the most frequently used verb for English was the general verb “put” for both non-signers (69%) and L2 signers (63%). The general placement verbs, “move” and “place” were the also among the most frequently used, and two general verbs “be” and “bring” were used infrequently. Four specific verbs occurred: “hang,” “drape,” “spread” (used only by non-signers), and “stick” (used only by L2 signers). Two specific posture verbs, “set,” and “lay” also occurred in the data. “Set” occurred in both groups and “lay” only occurred in the L2 signer group. Thus, the specificity in verbal expression is very similar across non-signers and L2 signers. Overall, the native English speakers (non-signers and L2 signers) exhibited a pattern of verb use that is congruent with previous research, namely by preferring non-specific placement verbs (“put,” “move,” and “place”) at a mean rate of 85% for both the non-signers and L2 signers, and by specifically preferring “put.” A mixed effects logistic regression analysis of verb type (general vs. specific) revealed no difference between groups (ß = 0.085, *p* = 0.866).

**Table 5:** Verb frequencies in English by non-signers and L2 signers.

	**Verb type**	**Non-signers**		**L2 signers**	
		**Mean% (*N*)**	**SD**	**Mean% (*N*)**	**SD**
Put	General	0.69 (129)	0.17	0.63 (120)	0.24
Move	General	0.09 (16)	0.04	0.14 (27)	0.18
Place	General	0.07 (13)	0.18	0.08 (15)	0.12
Hang	Specific	0.07 (14)	0.04	0.06 (11)	0.04
Set	Specific	0.06 (11)	0.09	0.04 (8)	0.08
Be	General	0.02 (3)	0.04	0.00 (0)	-
Drape	Specific	0.01 (2)	0.03	0.02 (3)	0.03
Bring	General	0.01(2)	0.02	0.00 (0)	-
Spread	Specific	0.01 (1)	0.01	0.00 (0)	-
Stick	Specific	0.00 (0)	-	0.02 (4)	0.04
Lay	Specific	0.00 (0)	-	0.01 (2)	0.02

#### English Co-speech Gesture

We next analyzed the co-speech gestures in the English data from the non-signers and the L2 signers. First, we asked whether exposure to ASL led the L2 signers to produce more iconic gestures (including gestures representing the Figure object, Path only, and Ground only, see Methods) in English compared to the non-signers. As a group, the non-signers produced a total of 185 gestures (M = 24.71, SD = 5.84) during their placement descriptions and the L2 signers produced a total of 121 gestures (M = 15.13, SD = 13.81). Thus, it was not the case that the L2 signers gestured more than the non-signers. A Poisson regression modeling number of gestures as a function of group showed no significant difference between the groups (L.R. χ^2^ = 1.915, *df* = 1, *p* = 0.1881)^11^.

Next, we asked whether there is evidence of bidirectional transfer in the L2 signers' English co-speech gestures. It is possible that the L2 signers' semantic categories have realigned with ASL. This is because they have acquired from the ASL system the use of classifiers to make distinctions between different placement events (even if they do not always use the appropriate handshape). There was no evidence of realignment in speech. However, as discussed, English has only a few specific placement verbs. This, together with the fact that the specific placement verbs have low frequencies makes it unlikely that L2 signers would use them as a prominent part of their inventory of placement expressions. For this reason, speech data alone may not accurately reflect the state of the L2 signers' semantic organization, but English co-speech gesture data may.

**Figure 6:**
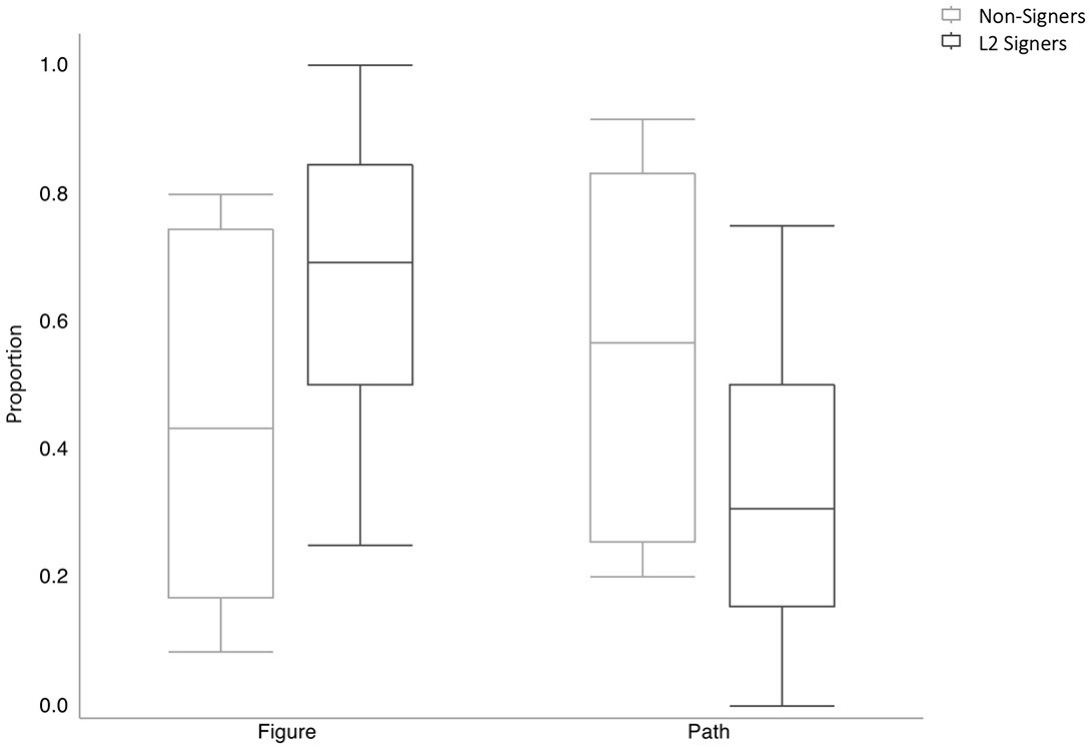
Proportion gesture type in English by group.

If there is bidirectional transfer, then the co-speech gestures of L2 signers should look different than those of non-signing English speakers. Specifically, we expect L2 signers to produce more gestures incorporating figure object information and less path-only gestures compared to non-signers. To assess whether this was the case, we focused on gestures that expressed information about Path only vs. about Figure. One L2 signer produced no Figure or Path gestures and was excluded from this analysis. We compared non-signers' and L2 signers' use of these gesture types (Figure 6). A mixed effects logistic regression analysis of gesture type as a function of group revealed a marginally significant effect of group (ß = −1.256, *p* = 0.065), with L2 signers' producing numerically more gestures about figure than path-only gestures (67% vs. 33%, SD = 26) compared to the non-signers (46% vs. 54% SD = 28).

Given that the L2 signers frequently used classifiers in their ASL descriptions, it is possible that their distribution of gesture types is affected by ordering effects in the experiment. Specifically, half the L2 signers completed the task in ASL before completing it in English. Thus, the L2 signers' higher rate of figure gestures could be a result of priming from ASL. We compared the distribution of gesture types between the L2 signers who did the task in ASL first and those who did the task in English first. If the higher rate of figure gestures in the bilingual group as a whole is driven by priming effects in the ASL-first participants, then we should see higher rates of figure gestures in that group compared to the English-first group. This was not the case. The mean rate of figure gestures was 54% (SD = 24) for the ASL-first group (*N* = 4) and 85% (SD = 15) for the English-first group (*N* = 3). A mixed effects logistic regression analysis revealed no significant difference between those L2 signers who completed the task in English first and those who completed the task in ASL first (ß = −1.012, *p* = 0.184).

## Discussion

The present study examined placement descriptions by second language (L2) learners of American Sign Language (ASL) and asked two questions, namely (1) whether learning the semantics of placement descriptions in a typologically different L2 in a different modality presents a challenge similar to when it occurs within the same modality, and (2) whether cross-modal L2 learners show evidence of semantic reorganization in placement descriptions in their first language (L1).

We found that the hearing L2 ASL signers used verb types similarly to Deaf, native signers and even incorporated classifiers at a comparable, if still somewhat lower, rate. Based on previous research, we would predict that the L2 signers in the present study should have problems acquiring the ASL placement verb system, given its complexity compared to English. Specifically, we expected non-target like use of classifiers suggesting fuzzy semantic boundaries. It is then perhaps surprising to find that the hearing ASL learners were well on their way to acquiring native-like placement descriptions. This is not to say that L2 signers are fully target-like in their ASL use. Classifier handshape ratings from a Deaf, native signer were lower for the L2 signers than for native signers, suggesting that the L2 learners were less ASL appropriate in their handshape selection. Moreover, the L2 signers used a wider variety of handshapes than the native signers, which is in line with previous findings suggesting that L2 signers tend to struggle with selecting target-like handshapes for objects and make distinctions that are too fine-grained (Schembri et al., 2005; Brentari et al., 2012; Marshall and Morgan, 2015; Janke and Marshall, 2017). Finally, the present study focuses on the semantics of placement verbs. It is likely that an investigation of the syntactic constructions and pragmatic contexts in which placement verbs participate would reveal additional differences between L2 learners and native signers.

Nevertheless, the L2 signers in the present study performed unexpectedly similar to Deaf, native signers with respect to including figure information in placement verbs. This is so especially in light of the learners' limited ASL experience, which was <3 years on average (mean 2.88 years, range 1–8 years). By comparison, English learners of Dutch who were residing in the Netherlands and therefore immersed in the language and culture for several years on average (mean length of residence: 11 years, range: 4 months to 19 years) show substantial problems with Dutch placement verbs (Gullberg, 2009a). However, this difference is not necessarily attributable to the difference in modality *per se*. First, it is a limitation of the present study that we only had two Deaf signers in the control group for ASL. With additional signers, clearer patterns in classifier preference for placement of different object types could emerge and possibly a statistically significant difference in classifier rate between groups. The ASL L2 learners' relative success could also be due to the higher semantic transparency in ASL verbs compared to a language like Dutch. For example, in a study of placement verb acquisition in Tamil and Dutch speaking children, Narasimhan and Gullberg (2011) found that Tamil children acquire relatively infrequent placement verbs (specifically caused posture verbs) early. They attribute this to the semantic transparency of Tamil verbs, which consist of multi-morphemic units such as “make stand” that “individually label the causal and result subevents” (2011, p. 504). By comparison, Dutch caused posture verbs are highly frequent but are also monomorphemic and much less transparent. ASL placement verbs are highly transparent, consisting of a root verb movement (e.g., “MOVE”) combined with a classifier representing either the handling or the shape and size of an object. It is possible that ASL learners are capitalizing on this transparency.

Another possibility is that L2 signers are benefitting from the fact that there is some overlap between the distinctions used in English co-speech gesture and in ASL. Specifically, even though native English speakers predominantly gesture about path in their English, they also gesture about the figure object at a non-negligible rate (46% in the present study, and 40% in Hoetjes, 2008). This overlap may help L2 signers reach the rate of 56% incorporation of classifiers expressing figure object information in ASL observed in the present study sooner than expected given their proficiency.

Regardless of the underlying reason, the results of the present study suggest that ASL learners successfully begin to reorganize their placement verbs semantics in the context of using ASL. It then becomes an important question whether this reorganization happens independent of their English semantics, that is whether their original system is still intact or whether the ability to express new and additional placement distinctions in ASL results in more far-reaching semantic changes. Previous work has found that same-modality learners can exhibit prolonged maintenance of their native placement distinctions, to the extent that L1-like patterns can be observed in L2 gesture, even when speech has become target-like (Gullberg, 2009a; Hoetjes, 2018). This suggests a persistence of L1 semantic organization for the purposes of speaking in the L2. In the case of ASL L2 acquisition, it is not possible to directly examine L2 gestures co-occurring with a main (verbal) expressive channel to assess whether there is evidence of maintenance of L1 semantics. However, the fact that L2 signers included figure information at a rate comparable to Deaf native signers along with the evidence of bidirectional transfer from the L2 to the L1 in the L2 signers' co-speech gesture pattern suggests limited persistence of L1 semantic patterns.

Unlike previous work (Weisberg et al., 2020), we did not find the cross-modal L2 learners in this study to use more iconic gestures than non-signers. This result is possibly an effect of proficiency and length of signing experience. In the present study, the L2 signers had around 3 years of sign experience on average. The L2 signers in the study by Weisberg and colleagues had 10 or more years of exposure to ASL, suggesting that increased gesturing may occur with increased exposure to and proficiency in ASL. In support of this hypothesis, Casey et al. (2012) found no clear increase in co-speech gesture frequency in L2 learners after 1 year of academic ASL instruction. While they found a numerical increase in gesture rate in the different-modality learners that was absent from the control group of same-modality language learners, the learner groups did not differ statistically from each other in terms of gesture rate. In the present study, however, we found the non-signers to use numerically (although not statistically significantly) more iconic gestures than the L2 learners. This difference could be due to the focus in the present paper on analyzing only the subset of participants' utterances that pertained to how an object was placed. Such utterances occur in specific discourse contexts, where the figure object is typically known information. Previous work has shown discourse context to affect gesture rate in non-signers (Debreslioska and Gullberg, 2020). We leave it for future research to determine whether L2 signers' and non-signers' gesture rates vary in a similar or different manner as a function of discourse context.

However, gesture rate was not the only domain we examined for bidirectional language transfer. In assessing the type of gestures used, we found that L2 signers gestured about the figure object (numerically) more than native English speaking non-signers, although statistically, the difference was only a trend. Nevertheless, the results suggest that on average the L2 signers have begun diverging from the monolingual pattern of gesturing nearly equally about Figure and Path and are instead favoring gestures about the figure object. The results here suggest that L2 learners' original system is not intact. This is remarkable given their profile as adult L2 learners of intermediate proficiency, with an average of <3 years of experience. This finding therefore raises questions about how gesture patterns compare in the native language of monolinguals and bilinguals when L2 learning happens within modalities. While previous work on placement descriptions has mainly focused on performance in the L2, one analysis of L2 learners' gesture patterns in the L1 replicated results for monolinguals in the same language (compare Hoetjes, 2008; Gullberg, 2009a). While more work is needed to confirm these patterns, comparing the L1 results of the current study to those of previous work suggests that semantic boundaries may be differentially affected by the L2 early on in acquisition in same- vs. different modality learning. In this context, it will be especially important to compare the present findings to situations in which both the L1 and the L2 are spoken and the L2 has specific placement verbs with transparent semantics in order to assess the role of a high degree of semantic transparency vs. the modality of the second language.

## Conclusion

This study examined object placement event descriptions by English L1-ASL L2 language users, asking whether cross-modal L2 learners face similar challenges to same modality L2 learners in learning to talk about placement. Placement verbs in signed languages such as ASL tend to be highly iconic and to exhibit transparent semantic boundaries which could facilitate their acquisition. We also asked how exposure to a typologically different language affects different semantic boundaries in placement events in the L1. Overall, L2 signers used ASL placement descriptions that looked similar to the Deaf, native signers', despite using a wider range of classifier handshapes and using them less appropriately, indicating somewhat fuzzy and less target-like boundaries in their placement semantics. Moreover, the L2 signers' English co-speech gesture patterns suggest that learning ASL may affect conceptualization of placement in the L1. Specifically, the placement distinctions expressed in co-speech gesture by the L2 signers were marginally more ASL-like compared to non-signers' gestures. Taken together, these results suggest that the iconicity and transparency of placement distinctions in the visual modality may facilitate semantic reconstruction in the placement domain, leading to increased target-like use of placement distinctions in the L2 as well as L1 placement distinctions that may differ from those of non-signers with the same first language.

## Data Availability Statement

The raw data supporting the conclusions of this article will be made available by the authors, without undue reservation.

## Ethics Statement

The studies involving human participants were reviewed and approved by Human Research Protections Program, UC San Diego. The participants provided their written informed consent to participate in this study. Written informed consent was obtained from the individual(s) for the publication of any potentially identifiable images or data included in this article.

## Author Contributions

AF conceived and designed the study, conducted analyses, and wrote the manuscript. Data collection and coding was carried out with the help of research assistants.

## Conflict of Interest

The author declares that the research was conducted in the absence of any commercial or financial relationships that could be construed as a potential conflict of interest.

## Publisher's Note

All claims expressed in this article are solely those of the authors and do not necessarily represent those of their affiliated organizations, or those of the publisher, the editors and the reviewers. Any product that may be evaluated in this article, or claim that may be made by its manufacturer, is not guaranteed or endorsed by the publisher.

## Footnotes

^1^“MOVE” and “PUT” both use the same flattened O handshape in the citation form (see Supplemental Images). The two verbs differ in their movements: whereas “PUT” has a defined ending point only, “MOVE” has both a defined beginning and ending point.

^2^Although note that it is at present an empirical question whether classifier incorporation is generally obligatory in placement descriptions.

^3^Spanish is similar to English in using general rather than specific placement verbs. Therefore, we do not expect the simultaneous speech-speech bilinguals to talk about placement differently than other English-speaking non-signers.

^4^Pilot work showed that this list was necessary in order for the participants to remember and describe every placement event. Unlike previous studies (e.g., Gullberg, 2009a), we used a list of pictures rather than written words in an effort to stay consistent between languages, as ASL has no conventionally recognized written form.

^5^“*SET”* is excluded from the table. Its citation form is made with an A-handshape that is sometimes considered a classifier and can be used to represent placement or location of objects, but this handshape does not alternate with other classifiers. We opted for a conservative analysis that does not treat instances of “*SET”* as containing a classifier that represents the figure object. However, an analysis that includes all instances of “*SET”* as having classifiers also does not result in any between-group differences.

^6^ASL has no copula. Intransitives are descriptions such as “PLATE ON TABLE,” “the plates are on the table”.

^7^Mixed effects logistic regression models were fit in R Core Team (2014) using the lme4 package (Bates et al., 2014). Models included random effects of subjects and items. Coefficient estimates, and *p*-values based on the Wald Z statistic are reported.

^8^Excepting the Fabric and Other categories, these categories largely correspond to the object shapes discussed by Zwitserlood (2012).

^9^For the Fabric category, the native signers used a large number of handshapes that were appropriate for showing how fabric items are handled but these handshapes were indistinguishable from citation forms and were consequently not counted as classifiers, as explained in the methods.

^10^Ordinal mixed effects models were fit in R Core Team (2014) using the packages Ordinal (Christensen, 2019), Car (Fox and Weisberg, 2019), and RVAdeMemoire (Hervé, 2021). Models included random effects of subjects and items.

^11^We used a generalized linear model for this regression. Because over-dispersion was indicated, we opted to use a quasi-poisson regression model.

## Supplementary Material

Supplementary Image 1 MOVE.

Supplementary Image 2 PUT.

Supplementary Image 3 HANG.

Supplementary Image 4 DRAPE.

Supplementary Image 5 Intransitive construction without classifier: the bag is on the door.

Supplementary Image 6 Intransitive construction with classifier: the magazine is on the table.

Supplementary Image 7 SET.

Supplementary Image 8 5 handshape (handling).

Supplementary Image 9 A handshape (handling).

Supplementary Image 10 C handshape (handling).

Supplementary Image 11 C handshape, flat (handling).

Supplementary Image 12 O handshape (handling).

Supplementary Image 13 S handshape (handling).

Supplementary Image 14 B handshape (entity).

Supplementary Image 15 Y handshape (entity).

Supplementary Image 16 F Handshape (entity).

Supplementary Image 17 C handshape, flat (entity).

Supplementary Image 18 baby-C handshape (entity).
