# Supplementary figures and images for: Emerging ASL Distinctions in Sign-Speech Bilinguals' Signs and Co-speech Gestures in Placement Descriptions
Source: Front Psychol. 2021 Aug 3;12:686485. doi: 10.3389/fpsyg.2021.686485 (PMC8369348; doi:10.3389/fpsyg.2021.686485)

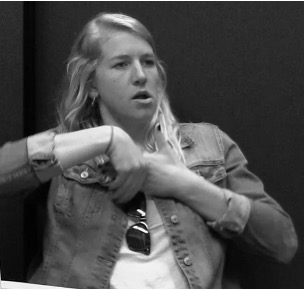

Supplement: Supplementary Image 1 — MOVE. [file Data_Sheet_1.zip › Supplemental Images/Image 17.jpg]

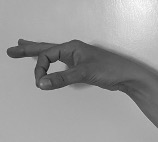

Supplement: Supplementary Image 1 — MOVE. [file Data_Sheet_1.zip › Supplemental Images/Image 16.jpg]

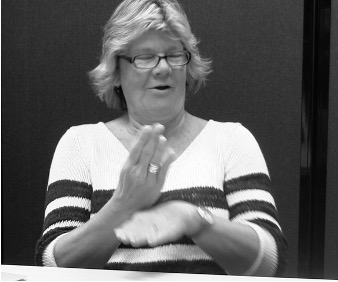

Supplement: Supplementary Image 1 — MOVE. [file Data_Sheet_1.zip › Supplemental Images/Image 14.jpg]

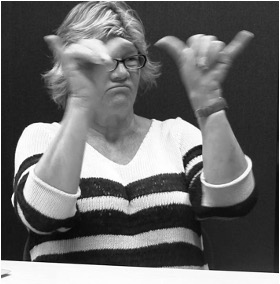

Supplement: Supplementary Image 1 — MOVE. [file Data_Sheet_1.zip › Supplemental Images/Image 15.jpg]

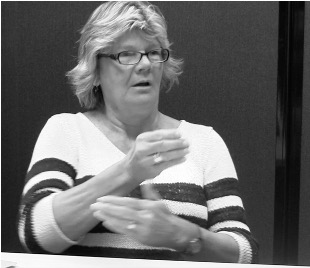

Supplement: Supplementary Image 1 — MOVE. [file Data_Sheet_1.zip › Supplemental Images/Image 11.jpg]

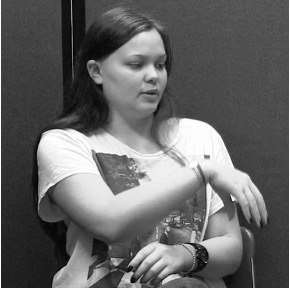

Supplement: Supplementary Image 1 — MOVE. [file Data_Sheet_1.zip › Supplemental Images/Image 8.jpg]

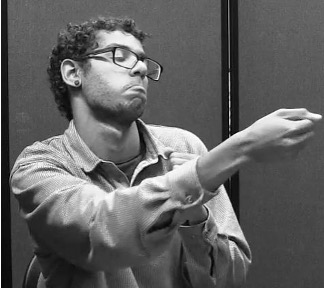

Supplement: Supplementary Image 1 — MOVE. [file Data_Sheet_1.zip › Supplemental Images/Image 9.jpg]

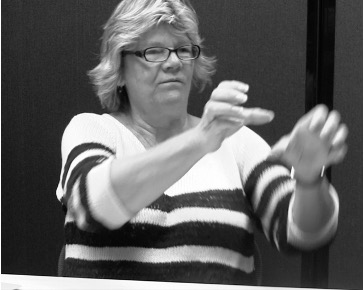

Supplement: Supplementary Image 1 — MOVE. [file Data_Sheet_1.zip › Supplemental Images/Image 10.jpg]

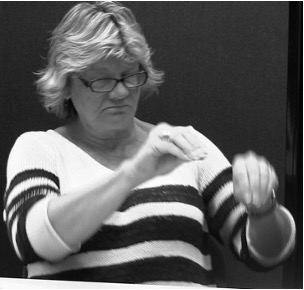

Supplement: Supplementary Image 1 — MOVE. [file Data_Sheet_1.zip › Supplemental Images/Image 12.jpg]

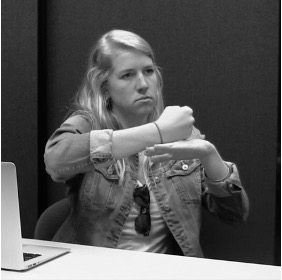

Supplement: Supplementary Image 1 — MOVE. [file Data_Sheet_1.zip › Supplemental Images/Image 13.jpg]

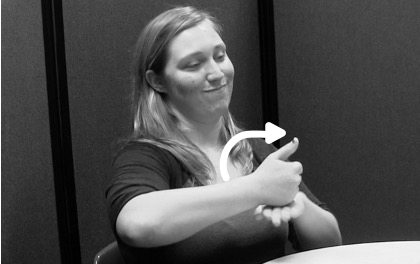

Supplement: Supplementary Image 1 — MOVE. [file Data_Sheet_1.zip › Supplemental Images/Image 7.jpg]

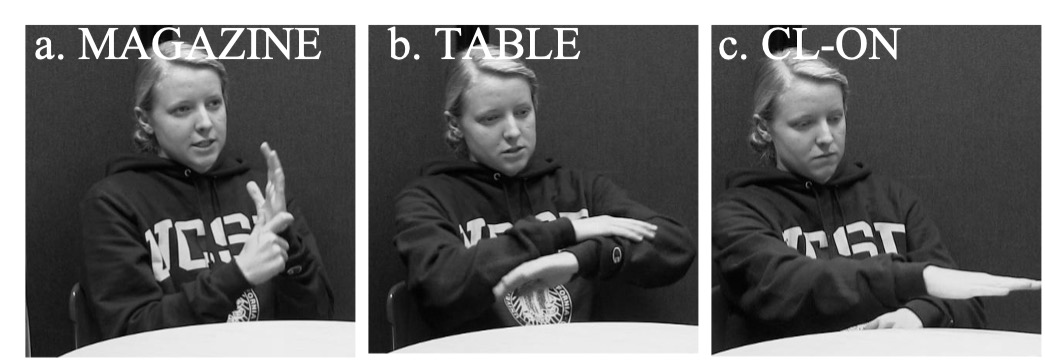

Supplement: Supplementary Image 1 — MOVE. [file Data_Sheet_1.zip › Supplemental Images/Image 6.jpg]

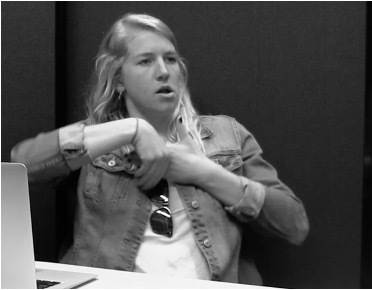

Supplement: Supplementary Image 1 — MOVE. [file Data_Sheet_1.zip › Supplemental Images/Image 4.jpg]

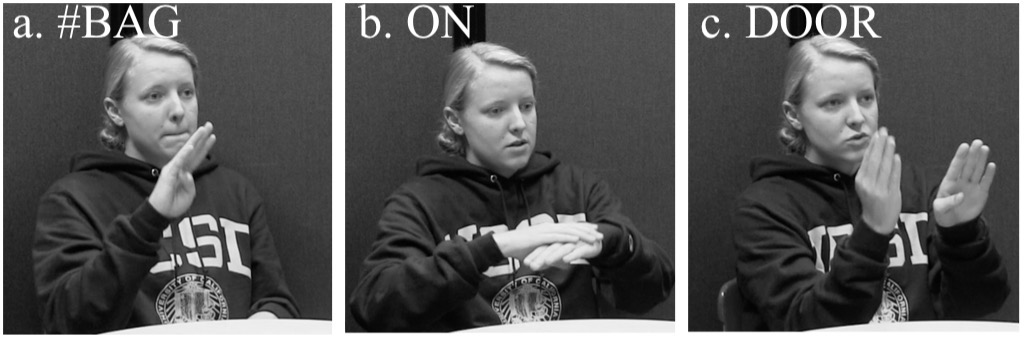

Supplement: Supplementary Image 1 — MOVE. [file Data_Sheet_1.zip › Supplemental Images/Image 5.jpg]

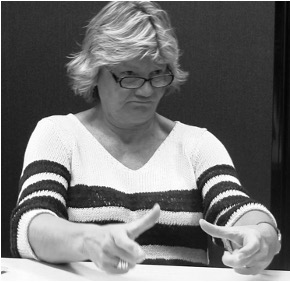

Supplement: Supplementary Image 1 — MOVE. [file Data_Sheet_1.zip › Supplemental Images/Image 18.jpg]

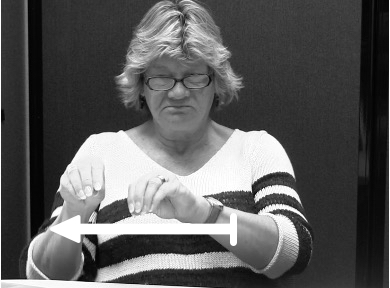

Supplement: Supplementary Image 1 — MOVE. [file Data_Sheet_1.zip › Supplemental Images/Image 1.jpg]

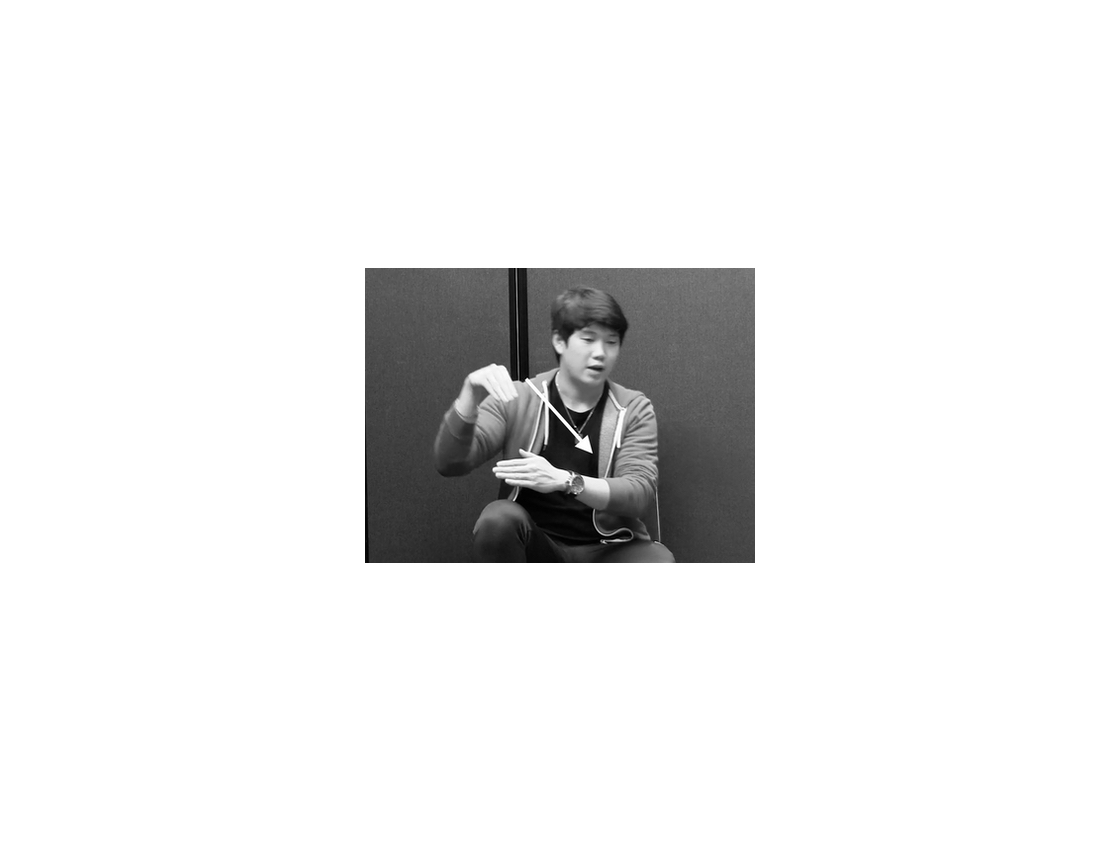

Supplement: Supplementary Image 1 — MOVE. [file Data_Sheet_1.zip › Supplemental Images/Image 2.jpg]

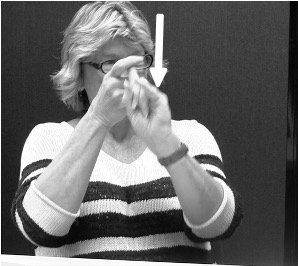

Supplement: Supplementary Image 1 — MOVE. [file Data_Sheet_1.zip › Supplemental Images/Image 3.jpg]
